# Determination of the median lethal dose of zinc gluconate in mice and safety evaluation

**DOI:** 10.1186/s40360-024-00736-8

**Published:** 2024-02-05

**Authors:** Yong-cai Wang, Xia Yang, Juan Xiao, Su-mei Wei, Ying Su, Xiu-qi Chen, Ting Huang, Qing-wen Shan

**Affiliations:** 1 Department of Pediatrics, The First Affiliated Hospital of Guangxi Medical University, No. 6, Shuangyong Road, 530021 Nanning, Guangxi Zhuang Autonomous Region China; 2 Guangxi Key Laboratory of Aquatic Genetic Breeding and Healthy Aquaculture, Guangxi Academy of Fishery Sciences, 530021 Nanning, China

**Keywords:** Zinc gluconate, Median lethal dose, Sub-lethal dose, Dose titration, Intravenous injection, C57BL/6J mice, ICP-MS

## Abstract

**Background:**

Zinc Gluconate (ZG) is a safe and effective supplement for zinc. However, there is limited research on the optimal dosage for intravenous injection and the safety evaluation of animal models for ZG. This study aims to determine the safe dose range of ZG for intravenous injection in C57BL/6J mice.

**Methods:**

A Dose titration experiment was conducted to determine the LD_50_ and 95% confidence interval (95%CI) of ZG in mice. Based on the LD_50_, four sub-lethal doses (SLD) of ZG were evaluated. Following three injections of each SLD and monitoring for seven days, serum zinc levels were measured, and pathological changes in the liver, kidney, and spleen tissues of mice were determined by histological staining.

**Results:**

The dose titration experiment determined the LD_50_ of ZG in mice to be 39.6 mg/kg, with a 95%CI of 31.8-49.3 mg/kg. There was a statistically significant difference in the overall serum zinc levels (H = 36.912, *P* < 0.001) following SLD administration. Pairwise comparisons showed that the serum zinc levels of the 1/2 LD_50_ and 3/4 LD_50_ groups were significantly higher than those of the control group (*P* < 0.001); the serum zinc level of the 3/4 LD_50_ group was significantly higher than those of the 1/8 LD_50_ and 1/4 LD_50_ groups (*P* < 0.05). There was a positive correlation between the different SLDs of ZG and the serum zinc levels in mice (rs = 0.973, *P* < 0.001). H&E staining showed no significant histological abnormalities or lesions in the liver, kidney, and spleen tissues of mice in all experimental groups.

**Conclusion:**

The appropriate dose range of ZG for intravenous injection in C57BL/6J mice was clarified, providing a reference for future experimental research.

## Introduction

Zinc is an important component of many enzymes in the body and has significant physiological functions and a wide range of pharmacological effects [1]. The dynamic balance of zinc is crucial for maintaining the structure and function of the intestinal mucosa. Zinc strengthens the mechanical barrier function of the intestine through tight intercellular connections and reduces cell permeability [2, 3]. Zinc deficiency can damage the intestinal barrier integrity, leading to intestinal leakage and allowing pathogenic bacteria and other antigenic substances in the gut to directly contact intestinal epithelial cells (IEC), resulting in sustained mucosal inflammation [4]. Exogenous zinc supplementation can alleviate intestinal inflammation and improve diarrhea and other symptoms [5], thus maintaining the integrity of intestinal mucosal barrier morphology and function [6].

In enzyme systems, zinc often acts as a structural and catalytic component, contributing to the functionality of diverse enzymes across various biological pathways. For instance, zinc is integral to the functionality of metalloenzymes involved in DNA and RNA synthesis, playing a central role in maintaining genomic stability [7, 8]. Furthermore, zinc is a key component of enzymes participating in immune responses, where it modulates signaling pathways critical for immune cell function and host defense [9]. The role of zinc in antioxidant defense mechanisms is equally noteworthy, as it acts as a cofactor for enzymes such as superoxide dismutase, safeguarding cells against oxidative stress. Additionally, zinc’s involvement in neuronal signaling, neurotransmitter release, and synaptic plasticity highlights its indispensable role in the central nervous system [10, 11].

Zinc gluconate (ZG) is a safe and effective zinc supplement that can improve intestinal injury when injected into animal models. Skalny et al. showed that supplementing with ZG can reduce the apoptosis of IEC and increase their proliferative ability, regulate intestinal flora, maintain intestinal integrity, improve immunity, and reduce the incidence of diarrhea [12]. In an animal model of sepsis, pre-treated with ZG significantly improved the survival rate of rats and increased zinc accumulation in the intestinal mucosa [13]. ZG can also promote intestinal health by regulating the growth and reproduction of intestinal microorganisms [14].

However, currently, there is limited research on the optimal dose of ZG and safety assessment in animal models with intravenous injection. Earlier literature reported that all mice died after intraperitoneal injection of 45.0 mg/kg (amount/body weight) of ZG; even doses below the median lethal dose (LD_50_), ranging from 12 to 15 mg/kg, induced significant morbidity or death in some mice [15]. There are also literature reports that repeated intraperitoneal injection of ZG at 4 mg/kg/d in rats resulted in clinical adverse reactions, such as local intolerance or growth retardation within several days [13, 16]. These results indicate that the dose of ZG varies widely, possibly due to different animal models and routes of administration. This lack of consensus makes it difficult to compare the Effect of ZG across experiments and limits the design and implementation of future studies. Therefore, this study aims to establish a C57BL/6J male mouse model for one-time tail vein injection of ZG, calculate the LD_50_ using the modified Coriolis method, and design four sub-lethal doses (SLD) below the LD_50_ based on the results of acute toxicity tests to evaluate the safety of ZG further.

The results of this study will contribute safety assessment data for ZG in animal models and provide a basis for rational drug design with the potential for future clinical applications. Moreover, it can also serve as a reference for evaluating the safety of other trace element supplements.

## Materials and methods

### Experimental animals

Healthy SPF-grade male C57BL/6J mice, aged 4–6 weeks, with an average body weight of 20 ± 2 g, were provided by the Experimental Animal Center of Guangxi Medical University (license number SCXK Gui 2020-0003). The Ethics Committee of Guangxi Medical University approved all animal experimental procedures. One week before the experiment, mice were acclimatized to the experimental environment and housed in a barrier-level animal room with controlled temperature (22–25 ℃) and relative humidity (50–70%) and a 12 h/12 h light/dark cycle. During the experimental period, mice were fed with SPF-grade growth and reproduction feed and had free access to food and water.

### Main reagents and drugs

Zinc gluconate (Analytical reagent; purity 98%; Macklin, Shanghai, China); ultra-pure sterile water (Leagene Biotechnology, Beijing, China); 0.22 μm microporous filter (Biofil, Guangzhou, China); 4% paraformaldehyde fixative solution (Servicebio, Wuhan, China); nitric acid (Sigma-Aldrich, Shanghai, China); 30% hydrogen peroxide (Leagene Biotechnology, Beijing, China).

### Main experimental instruments

KW-XXY mouse tail vein injection imaging system (Kew Basis, Nanjing, China); disposable sterile insulin syringe (U100; Kindly Group, Shanghai, China); inductively coupled plasma mass spectrometer (iCAP Q; Thermo Fisher, Germany); graphite digestion instrument (DS-360; Gdana, Guangzhou, China); upright white light photography microscope (Eclipse Ci-L; Nikon, Japan); fully automatic dehydrator (Leica ASP300S), semi-automatic slicer (Leica RM2245), fully automatic staining machine (TS5015; LEICA, Germany).

### Preparation of zinc gluconate solution

Zinc gluconate (ZG) was prepared as previously described [17]. Briefly, the Zn(C_6_H_11_0_7_)_2_ stock solution (9.11 mg/mL) was prepared by dissolving ZG in ultrapure sterile water. The stock solution was filter sterilized with a 0.22 μm filter and stored in the dark at 4℃. Experimental concentrations were obtained with the equal-volume dilution of the stock solution to ensure that mice from each dosage group received the same volume of ZG solution.

### Determination of the median lethal dose (LD_50_) of zinc gluconate

#### Administration route and volume

Mice were administered a single dose of 8 mL/kg (volume/body weight) via tail vein injection.

#### Dose titration experiments

The preliminary experiment was conducted using the up-and-down dose titration method to determine the maximum non-lethal dose (LD_0_) and the minimum lethal dose (LD_100_) of ZG in mice and to estimate the approximate lethal dose range of ZG from 0 to 100% [18, 19].

Twenty mice were randomly divided into four groups (five mice per group) based on their weight, injected with a single dose of ZG (70.0, 65.0, 35.0, or 24.0 mg/kg) via the tail vein, and their survival was monitored for 48 h. The inter-group ratio R was calculated as logI according to the formula I=(logLD_100_-logLD_0_)/(n-1), where n is the number of mice used in the experiment [20].

The LD_50_ was calculated using the modified Coriolis method [21]. Sixty mice were randomly divided into six groups of ten based on their body weight and treated with saline (Control; NC) or ZG (70.0, 53.8, 41.4, 31.8, or 24.4 mg/kg). The ZG doses tested were at 1.3 times intervals since the value I was found to be 0.115 in the above analysis.

#### Modified coriolis method calculation [21]

logLD_50_ = X_m_–i(∑$$ p$$–0.5), obtained by converting: LD_50_ = log^− 1^ [X_m_–i(∑$$ p$$–0.5)]

LD_50_’s 95% CI = log^− 1^ (logLD_50_ ± 1.96S_m_logLD_50_)

$${{\text{S}}_{\text{m}}}{\text{logL}}{{\text{D}}_{{\text{50}}}}\, = \,{\text{i}}\sqrt {\left( {\sum {p\, - \,\sum {p{\text{2}}} } } \right)\,{\text{/}}\,\left( {{\text{n}}\, - \,{\text{1}}} \right)} $$

X_m_: the logarithm of the maximum injection dose, i: the logarithmic dose difference between adjacent dose groups, ∑p: the total mortality rate of each dose group, S_m_: the standard error of LD_50_, p: the mortality rate (%), ∑p^2^: the square of the total mortality rates, and n: the number of animals in each group.

### Evaluation of safety and serum zinc level of sub-lethal dose ZG

#### Animal grouping and treatment

Forty mice were randomly divided into five groups of eight based on body weight and treated as follows: 0.9% saline (NC); group I (1/8 LD_50_): 5.13 mg/kg; group II (1/4 LD_50_): 10.26 mg/kg; group III (1/2 LD_50_): 20.52 mg/kg; group IV (3/4 LD_50_): 30.78 mg/kg.

The stock ZG solution was diluted as described above and administered via tail vein injection once on the first, third, and fifth days of the experiment. Mice’s body weights and behaviors were recorded on days 0, 2, 4, and 7. On day seven, 48 h after the last ZG injection, mice were anesthetized, blood was quickly collected retro-orbitally, and the liver, kidney, and spleen were dissected and fixed in 4% paraformaldehyde for 24 h. The whole blood samples collected in blood collection tubes without anticoagulant were allowed to stand at room temperature for approximately 1 h, allowing natural coagulation of the blood and complete contraction of the blood clot. Subsequently, the samples were centrifuged at 4℃ (4000 rpm/min for 10 min), and the separated supernatant obtained was the serum. The separated serum was then transferred to a clean, labeled centrifuge tube using a sterile pipette and stored in a -80℃ freezer for future use. The fixed tissues were dehydrated in increasing concentrations of ethanol, paraffin-embedded, and cut into 5 μm sections. For staining, sections were dewaxed in xylene, rehydrated in decreasing concentrations of ethanol, and stained with hematoxylin and eosin (H&E staining). Stained slides were cover-slipped and photographed with a light microscope.

#### ICP-MS

The first method of GB 5009.268–2016, “National Standard for Food Safety-Determination of Multiple Elements in Food,” was adopted [22]. Thawed serum (100 µL) was mixed with 2 mL nitric acid for 1 h, followed by digestion in a graphite digester (100 °C/2 h, 130 °C/2-4 h, 160 °C/4 h). The acid was then reduced to 0.5 ml at 180 °C, made up to 5 mL with water, mixed well, and measured by ICP-MS. A blank test with the reagents was also performed. Each sample was measured three times, and the results were expressed in mg/L. ICP-MS working parameters: input power 1250 W; nebulizer flow rate 1.058 L/min; cooler flow rate 14.0 L/min; auxiliary flow rate 0.8 L/min; peristaltic pump speed 40 r/min; sampling depth 5 mm; sampling cone aperture 1.1 mm; skimmer cone aperture 0.5 mm; concentric nebulizer; scan mode jump peak; each peak was measured three times [23].

### Statistical analysis

The data were analyzed using the SPSS 27.0 software and graphed using GraphPad Prism 9.0. Normally distributed continuous data were expressed as mean ± standard deviation (x ± s), and differences between multiple groups were compared using one-way analysis of variance (ANOVA). Non-normally distributed continuous data were expressed as median, and differences between multiple groups were compared using the Kruskal-Wallis H rank sum test. After Bonferroni correction, pairwise comparisons were conducted. Correlations were analyzed with Spearman correlation analysis. *P*<0.05 was considered statistically significant.

## Results

### Calculation of LD_50_ and 95% confidence interval

We estimated that the ZG doses that will induce 100% and 0% mouse mortality to be 70.0 and 24.0 mg/kg, respectively. The results show that all mice treated with 70.0 mg/kg died within 10 min of treatment, while there were no deaths in the 24.0 mg/kg treated group (Table 1). The other administered doses induced varying levels of lethality. From this result, we obtained an I value of 0.115, which translated to an inter-group ratio (R) of 1.30. Based on this, we used ZG doses of 70.0, 53.8, 41.4, 31.8, and 24.4 mg/kg (1.3 times intervals between doses) for the LD_50_ estimation.

**Table 1:** Survival of expected experiments in mice

ZG dosage (mg/kg)	Record of death cases	Number of surviving mice at 48 h
70.0	All died within 10 min of administration	0
50.0	3 mice died within 12 h of administration	2
35.0	1 mouse died within 12 h of administration	4
24.0	No deaths within 48 h of administration	5

#### General signs of ZG acute toxicity

**Fig. 1:**
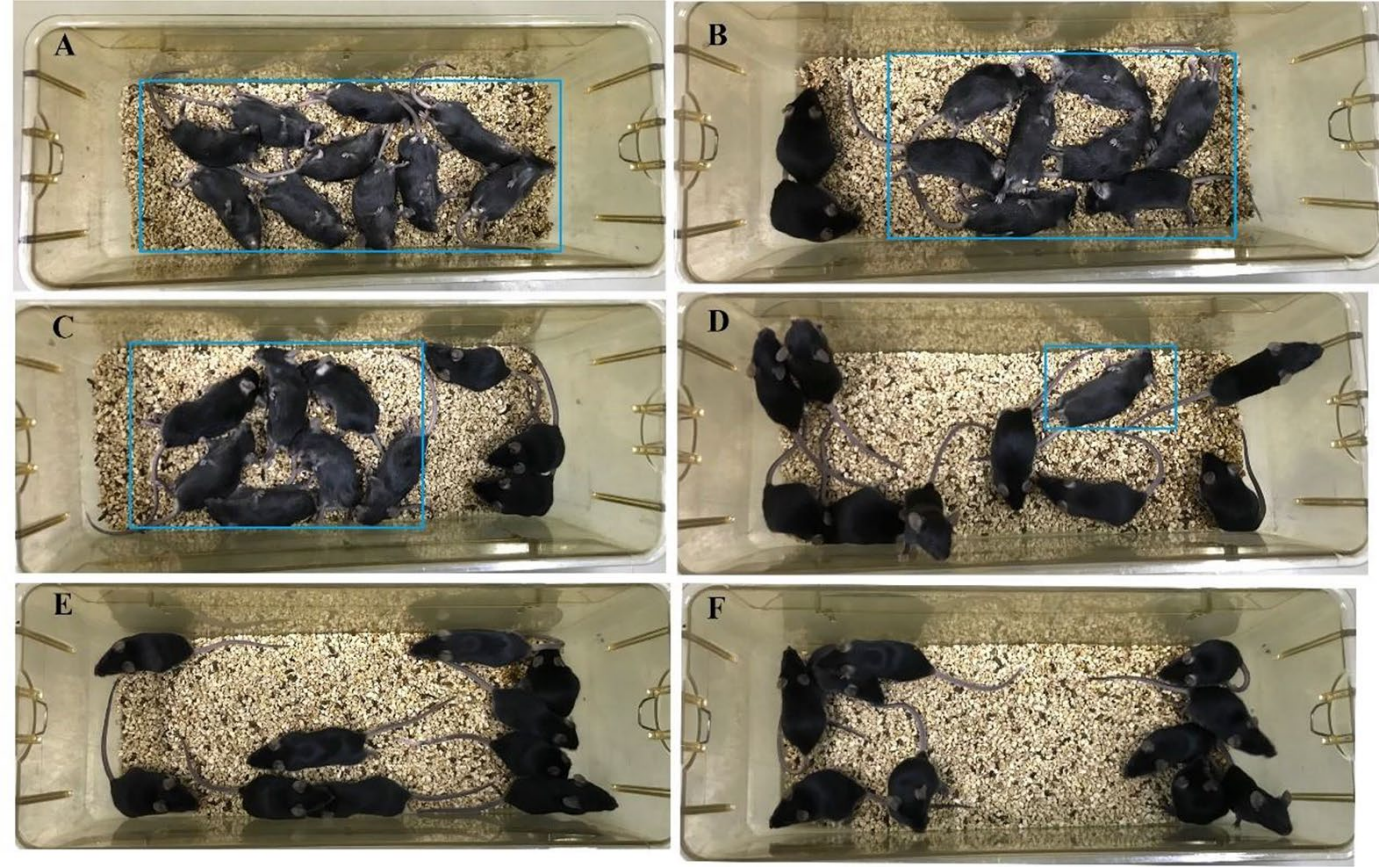
Acute toxicity and survival status of mice treated with (**A**) 70 mg/kg, (**B**) 53.8 mg/kg, (**C**) 41.4 mg/kg, (**D**) 31.8 mg/kg, and (**E**) 24.4 mg/kg of ZG, and (**F**) saline (control group). The rectangular box area marks the mortality of mice 12 h after the administration of ZG

After administration of the highest dose (70 mg/kg), mice quickly showed lethargy, limb weakness, prone or unsteady standing, lateral rotation, and forced convulsions and died within 10 min. After administration of 53.8 and 41.4 mg/kg, mice gradually showed reduced free activity, drowsiness, and semi-closed eyelids. When dying, they showed difficulty breathing and cyanosis. Most animals died within 12 h. The surviving mice were alive for the 48-hour observation window, and their mental state returned to normal. After administration of 31.8 mg/kg, a few mice showed a slight reduction in activity but quickly regained their vitality. One mouse died within 24 h, and the remaining surviving mice returned to normal during the 48-hour observation period. The clinical features of the lowest dose group (24.4 mg/kg) were consistent with the control group, with no abnormalities or deaths (Fig. 1).

#### Zinc gluconate LD_50_ and 95%CI

**Fig. 2:**
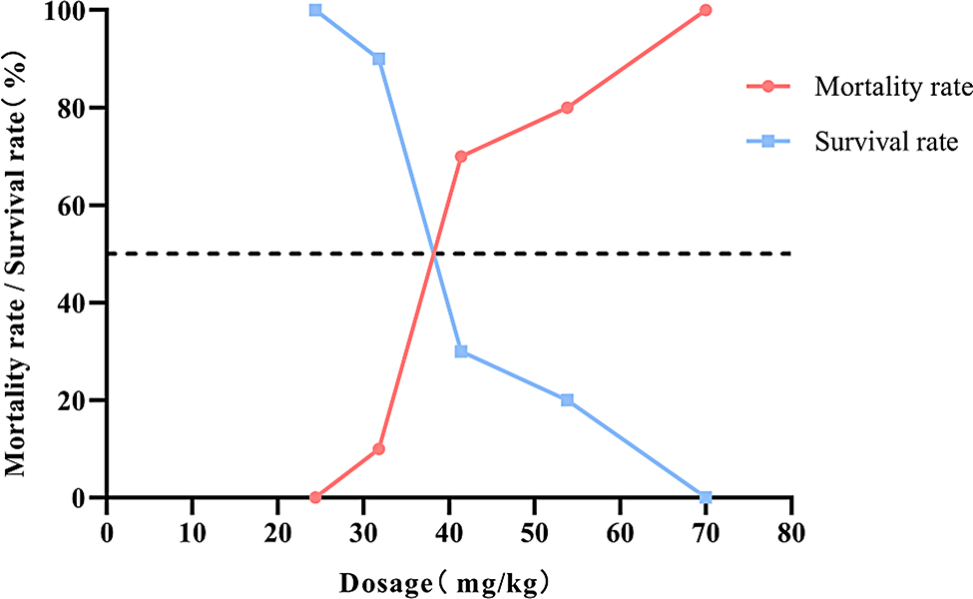
Dose-response curve of mortality/survival rate

The mortality rate and survival rate of mice treated with different doses of ZG are recorded in Table 2. From these data, we also plotted a dose-response curve of mortality rate/survival rate vs. dose (Fig. 2). According to the modified Coriolis method, the LD_50_ was calculated as 39.6 mg/kg, with a 95% CI of 31.8-49.3 mg/kg.

**Table 2:** Acute toxicity test results of ZG solution in mice

Dosage (mg/kg)	The logarithm of the dose	Number of mice	Number of deaths	Mortality rate (p,%)	Survival rate (q,%)	P^2^
70.0	1.85	10	10	100.0	00.0	1.00
53.8	1.73	10	8	80.0	20.0	0.64
41.4	1.62	10	7	70.0	30.0	0.49
31.8	1.50	10	1	10.0	90.0	0.01
24.4	1.38	10	0	00.0	100.0	0.00
00.0	-	10	0	00.0	100.0	0.00

### Safety assessment in mice treated with sub-lethal doses

#### Effect of zinc gluconate on body weight

**Fig. 3:**
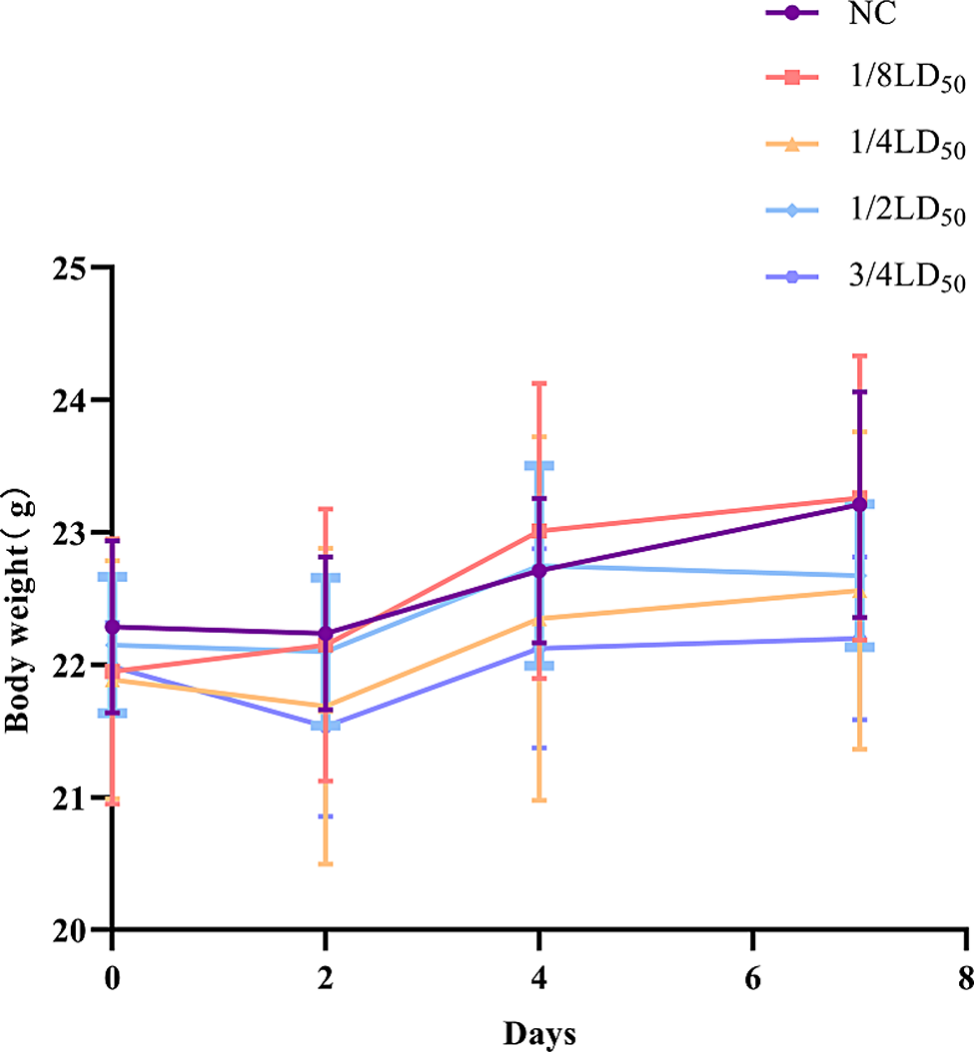
Effect of different sub-lethal doses of ZG on weight changes in mice

To determine whether sub-lethal doses (SLD) of ZG affect mice’s well-being, we injected mice three times for seven days with one of four different SLDs from 1/8LD_50_ to 3/4LD_50_ and monitored their body weight changes (Table 3; Fig. 3). Both the control and 1/8 LD_50_ groups showed a gradual increase in body weight over the seven days post-injection. All remaining ZG groups (1/4 LD_50_, 1/2 LD_50_, and 3/4 LD_50_) showed a slight decrease in body weight on the second day, followed by a gradual increase.

**Table 3:** Comparison of weight changes among mice treated with sub-lethal doses of ZG (x ± s)

Date	NC	I (1/8LD_50_)	II (1/4LD_50_)	III (1/2LD_50_)	III (3/4LD_50_)	F	P
Day 0	22.3 ± 0.6	22.0 ± 1.0	21.9 ± 0.9	22.2 ± 0.5	22.0 ± 0.3	0.409	0.801
Day 2	22.2 ± 0.6	22.2 ± 1.0	21.7 ± 1.2	22.1 ± 0.6	21.5 ± 0.7	1.070	0.386
Day 4	22.7 ± 0.5	23.0 ± 1.1	22.4 ± 1.4	22.8 ± 0.8	22.1 ± 0.8	1.081	0.381
Day 7	23.2 ± 0.9	23.3 ± 1.1	22.6 ± 1.2	22.7 ± 0.5	22.2 ± 0.6	2.042	0.110

As shown in Table 3; Fig. 3, we identified a noteworthy trend in the weight changes of mice within the experimental groups (1/4LD_50_, 1/2LD_50_, 3/4LD_50_) following ZG injection. A transient decrease in weight was observed, succeeded by a gradual and slow increase over time. In contrast, the control group and 1/8LD_50_ group exhibited a steady increase in mouse weight. We hypothesize that this pattern may be attributed to the promotive Effect of ZG on mouse weight at lower doses, while medium to high doses could induce temporary weight reduction before stabilization.

### Effect of zinc gluconate on major organs

At the end of the seven-day monitoring period, 48 h after the last ZG injection, we harvested the liver, kidney, and spleen for histological analysis.

#### H&E staining of the liver

**Fig. 4:**
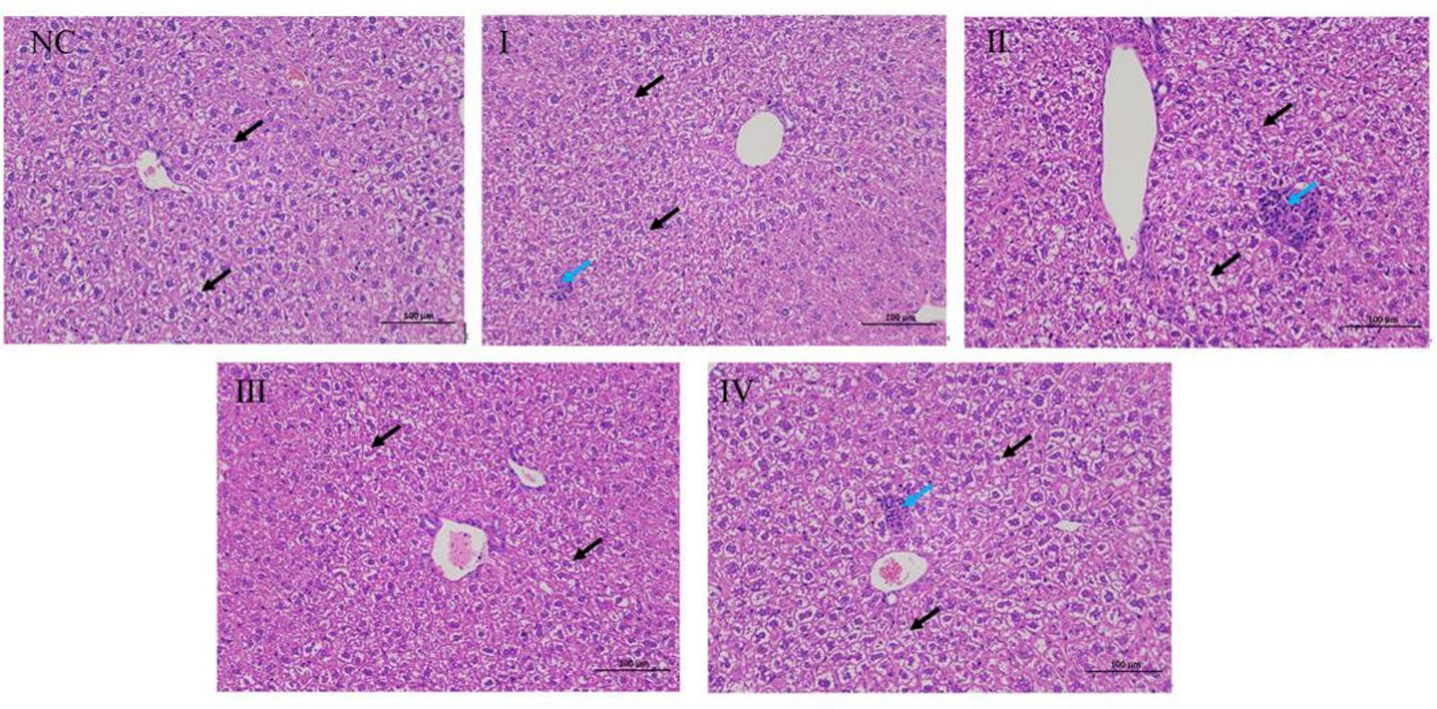
Histological analysis of the liver 48 h after the last intravenous injection of different sub-lethal doses of zinc gluconate. Representative images of H&E staining of liver sections. Black arrows indicate hepatocellular vacuolar degeneration and cytoplasmic vacuolization, and blue arrows indicate lymphocyte infiltration in the liver lobules. Magnification: x200. *Note*: NC:normal saline, I:1/8LD_50_,II:1/4LD_50_,1/2LD_50_,IV:3/4LD_50_

In the NC group, the membrane structure of the liver tissue was clear, and a few liver cells showed vacuolar degeneration and cytoplasmic vacuolization (black arrow). There was no obvious abnormality in the portal area and no obvious necrosis or inflammatory cell infiltration. In ZG intervention groups I (1/8LD_50_), II (1/4LD_50_), and IV (3/4LD_50_), a few lymphocytes were sporadically infiltrated in the liver lobules (blue arrow), and the remaining histological features were consistent with the NC group (Fig. 4).

#### H&E staining of the kidney

**Fig. 5:**
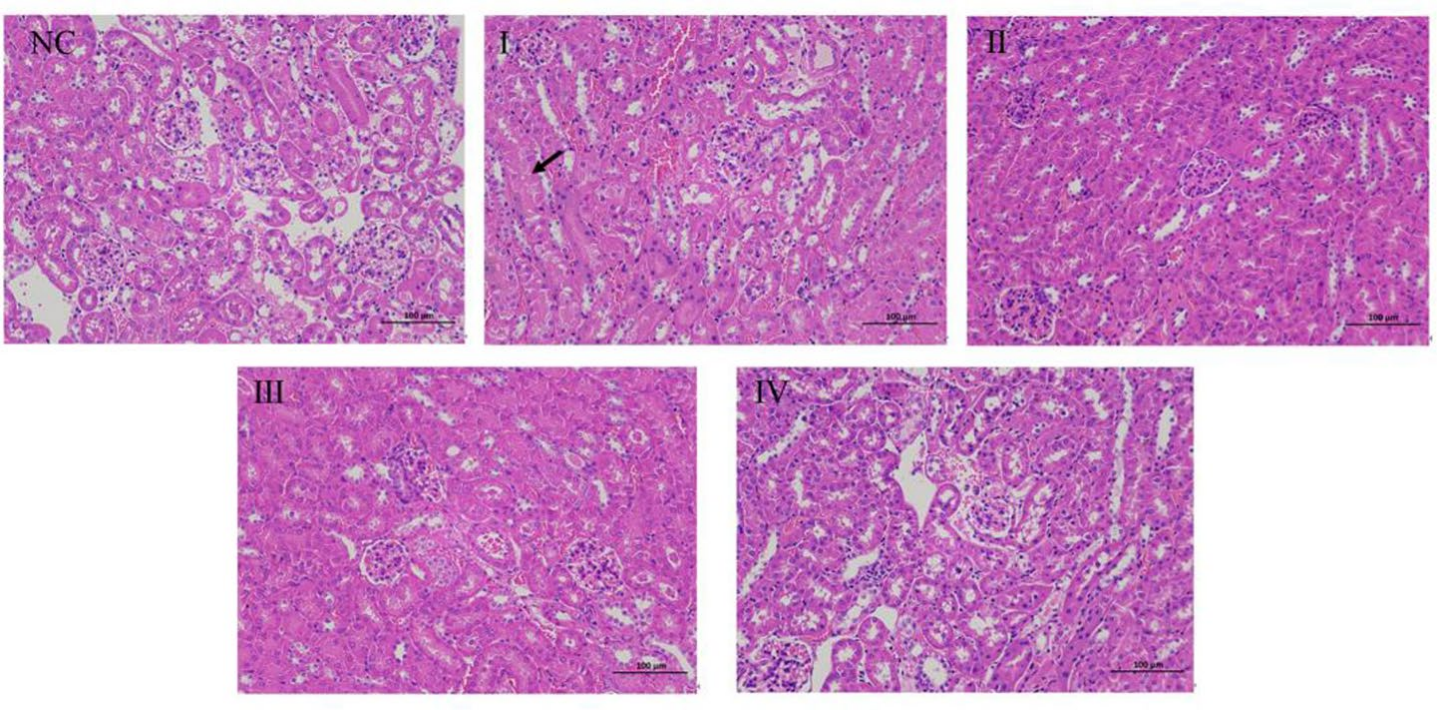
Histological analysis of renal tissue 48 h after the last intravenous injection of different sub-lethal doses of zinc gluconate. Representative images of H&E staining of kidney sections. The black arrow indicates renal tubular epithelial granular degeneration and cytoplasmic vacuolization. Magnification: x200. *Note*: NC:normal saline, I:1/8LD_50_,II:1/4LD_50_,1/2LD_50_,IV:3/4LD_50_

The structure of the glomerular basement membrane was clear in the renal tissue of the NC group. There were no significant pathological changes observed. The number of cells in the renal cortex and the cellular matrix was uniform, the epithelial cells of the renal tubules were round and plump, and the medulla showed no obvious abnormalities. The connective tissue between the urinary tubules, forming the renal interstitium, showed no obvious signs of proliferation. In the ZG intervention groups, slight granular degeneration of the renal tubular epithelial and cytoplasmic loosening was occasionally seen in group I (1/8LD_50_; black arrow), while groups II to IV were comparable with control (Fig. 5). Examining the various dosage groups under ZG intervention, occasional slight granular degeneration of renal tubular epithelia and cytoplasmic loosening were noted in the low-dosage Group I, indicated by a black arrow.

Conversely, the renal tissue morphology in the medium to high dosage Groups II to IV closely resembled that of the control group, exhibiting no discernible abnormalities. These findings prompted contemplation regarding the dosage-dependent effects of ZG. The mild tubular epithelial changes and cytoplasmic loosening in the low-dosage Group I may represent a specific biological response of renal tubular epithelia to ZG at lower concentrations, inducing subtle structural alterations. However, the renal tissue morphology in the medium to high dosage groups (II to IV) appeared largely unaffected, suggesting a minor or reversible impact of ZG on renal tissues at these concentrations. This similarity may support the assumption of relative safety associated with these dosage levels.

#### H&E staining of the spleen

**Fig. 6:**
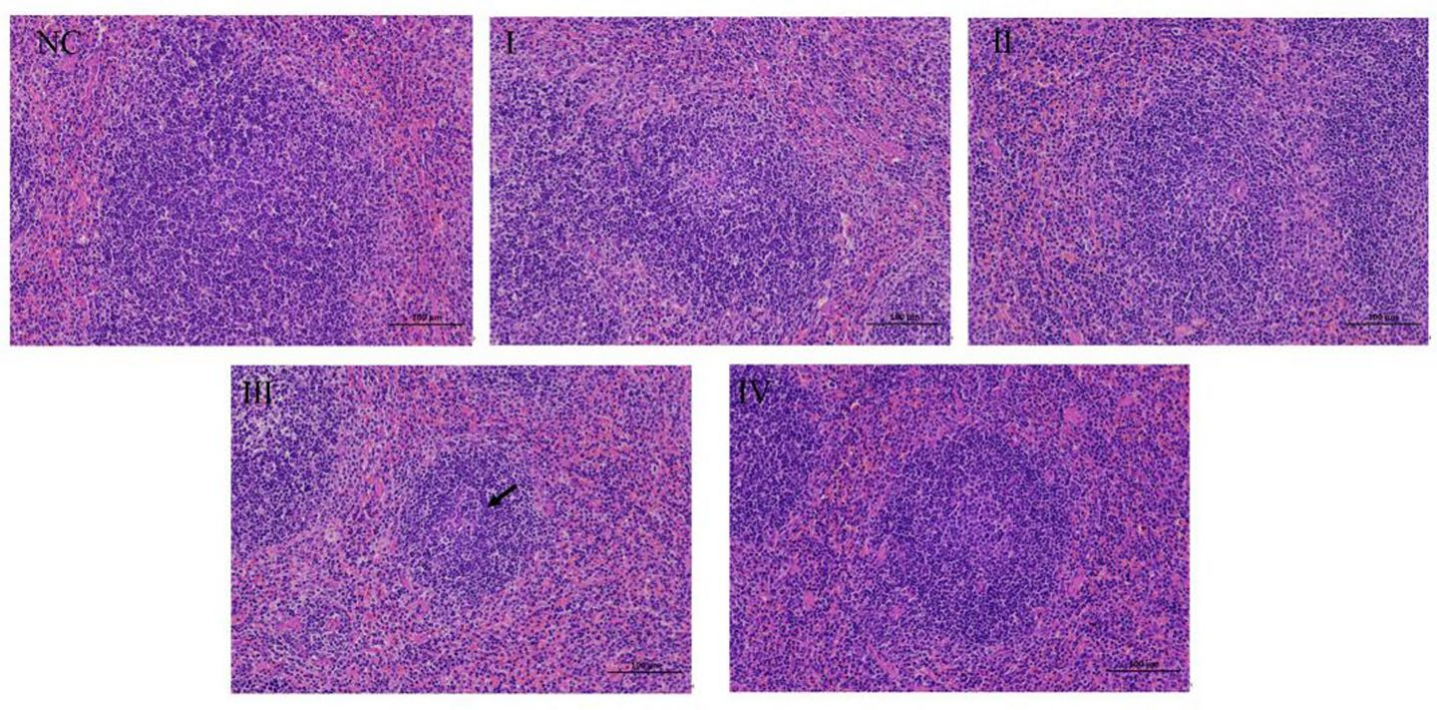
Histological analysis of the spleen 48 h after the last intravenous injection of different sub-lethal doses of zinc gluconate. Representative images of H&E staining of spleen sections. The black arrow indicates a decrease in the number of white pulp and a reduction of lymphocytes in local areas. Magnification: x200. *Note*: NC:normal saline, I:1/8LD_50_,II:1/4LD_50_,1/2LD_50_,IV:3/4LD_50_

Spleen tissue from the NC group showed clear membranous structures. The white pulp, marginal zone, and red pulp regions of the spleen can be easily distinguished with clear boundaries. The white pulp was abundant, with regular shapes, and was composed of dense lymphoid tissue. The marginal zone transitional region between the white and red pulp was present. The red pulp was distributed in a large area under the capsule, around the trabeculae, and at the periphery of the white pulp. It consisted of splenic cords and sinuses and was uniformly distributed without obvious pathological changes. In ZG group III (1/2LD_50_), there was a slight reduction in the amount of white pulp and occasional lymphocyte depletion in some areas (black arrow). The histological features of groups I, II, and IV were consistent with those of the NC group (Fig. 6).

### Effect of zinc gluconate on serum zinc levels in mice

**Fig. 7:**
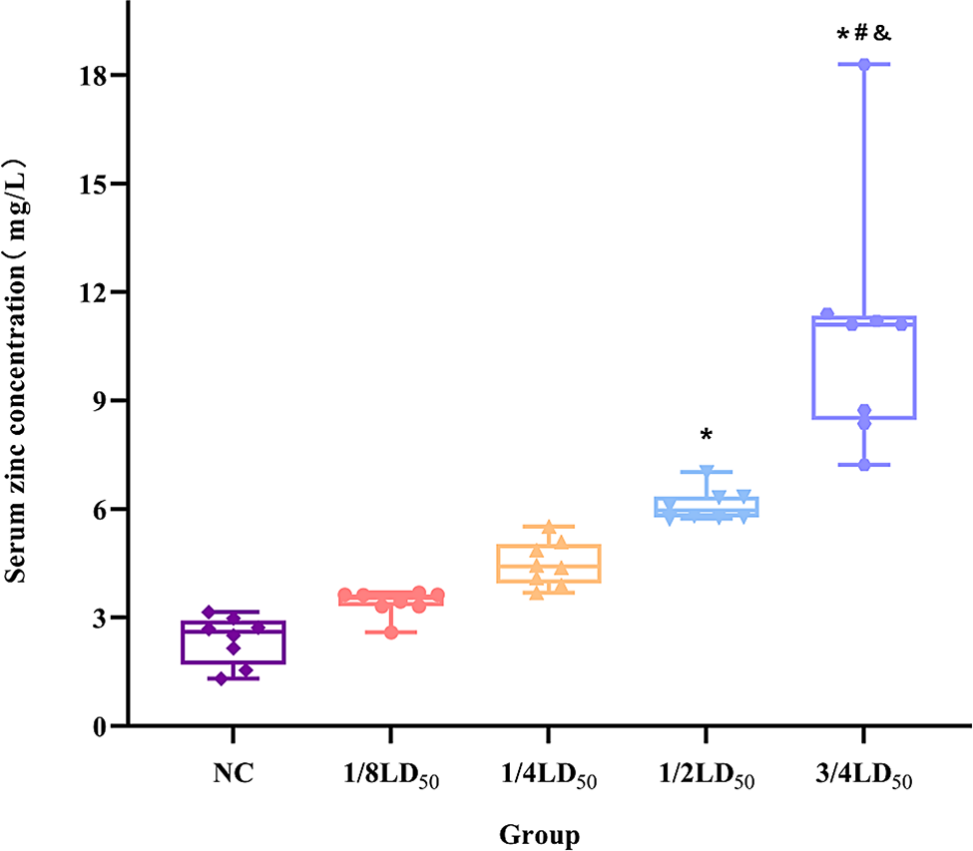
Effect of different sub-lethal doses of ZG on serum zinc levels in mice. *Note*: ^*^P < 0.001, compared with NC group; ^#^*P* < 0.05, compared with 1/8 LD_50_ group; ^&^*P* < 0.05, compared with 1/4 LD_50_ group

**Fig. 8:**
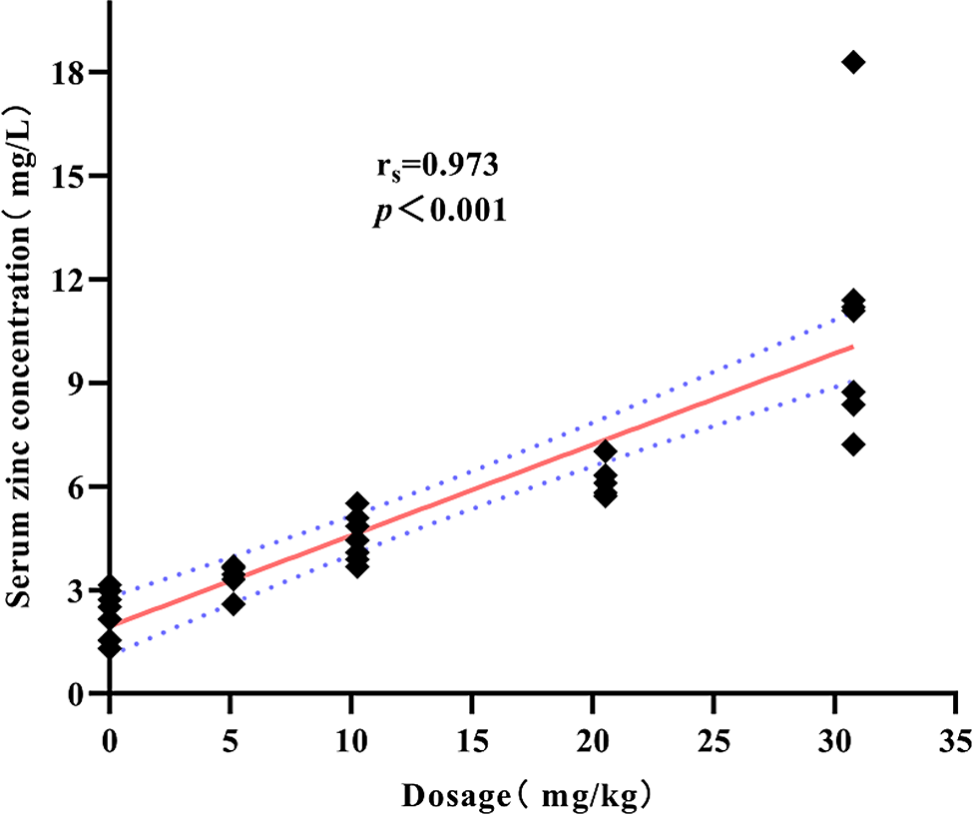
Correlation analysis between different sub-lethal doses of ZG and serum zinc levels in mice

To determine how intravenous injections of different SLD of ZG alter circulating zinc levels in mice, we quantified the serum zinc concentration on day seven, 48 h after the last injection of ZG (Fig. 7). ZG treatment significantly increased serum zinc levels (Kruskal-Wallis H test: H = 36.912, *P* < 0.001). After Bonferroni correction, pairwise comparisons showed that serum zinc levels in the 1/2LD_50_ and 3/4LD_50_ groups were significantly higher than control (*P* < 0.001). The serum zinc level of the 3/4LD_50_ group was also significantly higher than that of the 1/8LD_50_ and 1/4LD_50_ groups (*P* < 0.05). Spearman correlation analysis showed a positive correlation (r_s_ = 0.973, *P* < 0.001) between different SLDs of ZG and serum zinc levels in mice (Fig. 8). These results showed that serum zinc levels in mice increased as the injected zinc levels increased.

## Discussion

Zinc gluconate is a low-cost and easily obtainable drug that can be taken orally or used non-enterally [24]. A meta-analysis of multiple randomized controlled trials has shown that high-dose (45 mg/day) oral zinc supplementation did not significantly improve diarrhea, infection rates, or mortality rates and had no significant effect on serum zinc levels compared to the normal group [25, 26]. In addition, zinc has a strong metallic taste and unpleasant flavor and can cause vomiting when taken orally [27]. On the contrary, O’Kane et al. found that intravenous injection of a low dose of zinc (20 mg/kg) achieved a rapid 6-fold increase in serum zinc levels and significantly improved renal ischemia-reperfusion in sheep [28]. This suggests that intravenous injection can bypass intestinal absorption, eliminate factors that limit zinc bioavailability during oral administration by directly entering the bloodstream, and achieve optimal serum zinc levels, greatly increasing the drug’s bioavailability. In addition, for some patients with gastrointestinal injury, oral medication may stimulate the gastrointestinal tract and potentially antagonize the condition, while intravenous injection can avoid this stimulation and may be a more appropriate administration method.

The median LD_50_ represents the drug dosage under experimental conditions that cause the death of half of the tested animals. It is an indicator used to measure the Toxicity of substances [29]. A higher LD_50_ value indicates lower Toxicity of the drug and higher safety for the organism [30]. There are various methods for calculating LD_50_, among which Coriolis’s method is the most commonly used, the visual estimation of the probability unit method is the most convenient, and the sequential method utilizes the least animal. Calculations for the original Coriolis’s method are complex, tedious, time-consuming, and prone to errors. The method was subsequently improved upon by Tan et al. and is known as the modified Coriolis method [31]. The modified method requires the following conditions: drug doses must be divided into equal geometric groups; the number of animals in each group should be equal; dose groups with death rates of 0% and 100% must be included. The modified Coriolis’s method calculates directly according to the formula, without the need for data modification, and omits other complex processes. As a result, the calculation is quick and accurate.

Our study showed that the LD_50_ of ZG in mice (C57BL/6J, male, 4–6 weeks old) administered by tail intravenous injection was 39.6 mg/kg (95% CI: 31.8–49.3 mg/kg). There was a trend towards increased body weight over time in all groups, and there was no statistically significant difference between groups. These results indicate that the SLD of ZG has no significant effect on the body weight of mice (*P* > 0.05). The observed dosage-dependent Effect of ZG, where its impact on renal tissues varies with increasing dosage, forms the basis of our hypothesis. While low dosages may induce minor changes, the effects at medium to high dosages seem comparatively minor. The physiological significance of these research results may necessitate further exploration across additional ZG dosage levels to understand its effects on the biological organism comprehensively. Previous literature reported that all mice died after intravenous injection of 45.0 mg/kg, and even doses below LD_50_ (12–15 mg/kg) induced varying mortality rates [32]. The differences between these results and our study may be related to factors such as mouse species, solvent type, and experimental procedure. In our study, we adjusted the dose gradient and used the modified Coriolis method to calculate LD_50_, providing a more accurate dose basis for subsequent research. We observed that all mice died within 10 min of receiving the highest dose (LD_100_), indicating that 70.0 mg/kg is the absolute lethal dose of ZG for C57BL/6J male mice, while the lowest dose (LD_0_) at 24.4 mg/kg was safe with no deaths. For mice in each dose group above LD_0_, death mainly occurred within the first 12 h. A dose-response relationship was evident from the death rate/survival rate-dose curve.

SLD refers to a level below the LD_50_ of a substance or drug that can cause some reversible physiological or behavioral changes but does not result in death [33, 34]. In pharmacology and toxicology research, SLD can help researchers determine the therapeutic dose range of a drug, that is, the dose range that can produce a therapeutic effect without causing severe adverse reactions [35, 36]. It is worth noting that SLD is a relative concept that depends on factors such as the species, sex, age, and weight of the experimental animals, as well as the biological availability, Toxicity, metabolism, and excretion of the substance. The histology results of the main organs in this study showed that no significant abnormalities or lesions were observed in the liver, kidney, and spleen tissues with all SLD assessed. This is consistent with previous reports, including the work of Wang et al., which showed that ZG had no significant adverse effects on the major organs of Alzheimer’s disease (AD) mice and was an ideal organic zinc supplement with good biocompatibility [37–39]. C57BL/6J mice treated with intraperitoneal injections of ZG (30 mg/kg) continuously for three days did not show any toxic side effects. Andriollo-Sanchez et al. also found no significant adverse reactions in rats injected with a low dose (1-2 mg/kg/day) of ZG for a week [16]. In terms of physiological activity, appropriate doses (30 mg/kg) of ZG can increase G-CSF expression in microglia/macrophages through the NF-κB signaling pathway, promoting neuronal survival and functional recovery after spinal cord injury [40]. Choi et al. found that zinc at different SLDs can activate the phosphatidylinositol-3-kinase (PI3K) pathway to protect neural stem cells (NSCs) from hypoxic damage, thereby restoring NSC vitality and proliferation [41]. These results indicate that appropriate levels of ZG exhibit good biocompatibility and function with no significant toxic side effects. Indeed, appropriate doses of ZG have no adverse effects on human and animal health [42]. However, long-term high-dose intake of ZG may lead to reduced absorption of copper and iron [43]. Wang et al.found that ZG had no significant adverse effects on the major organs of AD mice at the injection dose and was an ideal organic zinc supplement with good biocompatibility, which is consistent with our experimental results [1, 44].

ICP-MS is a relatively new analytical technology developed in the 1980s [23, 45]. Compared to classical atomic absorption spectrometry, it has the advantages of being fast, simple, highly sensitive, low interference, and wide linear range. It has been widely used in geological research, food and medicine, environmental detection, and biomedical analysis [46].

In this study, ICP-MS was used to measure the zinc content in peripheral blood serum. The results showed that SLD dosage is positively correlated with serum zinc concentration. This is consistent with a previous systematic review and dose-response meta-analysis, which found that doubling zinc intake increases serum/plasma zinc levels by 9% [47, 48]. In addition, the correlation analysis showed that as the dosage of SLD increased, the serum zinc level also increased, which was consistent with the expected result and indicated a positive correlation between the dosage and serum zinc level [49]. Another study also demonstrated a positive linear relationship between blood zinc levels and the risk of non-alcoholic fatty liver disease (NAFLD) [50]. These results suggest that there is a dose-dependent relationship between injected ZG and serum zinc levels as more zinc enters the bloodstream with higher supplement doses. These findings may help guide the clinical application of zinc supplementation, such as in the treatment of zinc deficiency. Our study, conducted in a mouse model, provides valuable insights into the acute effects of ZG at different dosage levels. However, we acknowledge the inherent limitations of extrapolating these findings directly to clinical practice. The short observation period and the focus on peripheral serum zinc levels are notable constraints that need to be acknowledged.

Additionally, the chosen dosage levels may not fully capture the range of potential therapeutic or toxic effects associated with ZG. While our experiment offers a foundational understanding, we recognize the need for future research to validate these results in clinical settings. Expanding the study to include a broader dose spectrum, extending the observation period, and considering a more diverse range of organs for histopathological examination will contribute to a more comprehensive assessment of ZG’s safety profile and therapeutic potential.

## Conclusion

This study used a modified Coriolis method to calculate the LD_50_ of ZG administered via the tail vein in male C57BL/6J mice, which was found to be 39.6 mg/kg, with a 95% CI of 31.8 to 49.3 mg/kg. The safety of ZG in mice was confirmed by various SLD tests, providing a reference for subsequent experimental research work, as well as guidance for further related research and dosage form development of ZG. However, this study has certain limitations. Firstly, only the LD_50_ of ZG administered via the tail vein in male C57BL/6J mice was determined in this study, and the specific pharmacological effects and dose-response relationship of ZG were not further studied. Secondly, this study only evaluated the acute Toxicity and safety of ZG, and further research is needed to evaluate its long-term use and potential side effects.

## Data Availability

All data generated or analyzed during this study have been included in the published article files. Additionally, the datasets used and/or analyzed during the current study are available from the corresponding author upon reasonable request.

## Abbreviations

ZG: Zinc gluconate
LD_50_: Median lethal dose
SLD: Sub-lethal dose
95%CI: 95% confidence interval
ICP-MS: Inductively coupled plasma mass spectrometry
IEC: Intestinal epithelial cells

## Declarations

### ARRIVE guidelines statement

The authors have confirmed that the study was conducted in adherence to the ARRIVE guidelines.
